# Supplementary material for: Exploring Longitudinal Cough, Breath, and Voice Data for COVID-19 Progression Prediction via Sequential Deep Learning: Model Development and Validation
Source: J Med Internet Res. 2022 Jun 21;24(6):e37004. doi: 10.2196/37004 (PMC9217153; doi:10.2196/37004)
Supplement: Multimedia Appendix 1 [file jmir_v24i6e37004_app1.docx]

# Multimedia Appendix 1

## Data collection

A mobile app was developed and released in April 2020 in multiple languages and for multiple platforms (e.g., an Android app, an iOS app, etc.) to gather crowdsourced respiratory audio data. It collects the participants’ audio recordings, alongside their test results, demographics, medical history, and symptoms. The participants were asked to record three types of audio data: a cough recording with three cough sounds, a breathing recording with three to five inhalation-exhalation sounds, and a voice recording with participants repeating a sentence displayed on the screen three times. They were also asked to report their test labels, chosen from a positive test result, a negative test result, and an option of never being tested. Demographic data including age and gender was collected with participants’ permissions. Smoking history and other medical history such as asthma and high blood pressure were also collected. Participants were asked to report their symptoms chosen from a list of twelve types, including fever, chills, dry cough, wet cough, short of breath, tightness in your chest, headache, muscle aches, sore throat, runny or blocked nose, loss of taste and smell, and dizziness. No identifiable information was collected. The self-reported labels were adopted as the ground truth for model development and validation.

## Data selection

Positive participants are defined as those who have provided at least one audio recording alongside a positive test result, and negative participants are defined as those who have only ever tested negative. Since there are significantly more negative than positive participants, we first select the eligible positive participants to guarantee a relatively balanced dataset. We identified 168 positive participants who provide more than 4 samples, where the test results may also include negative (after recovery) and non-tested reports. Given the data was crowdsourced, we manually checked the label quality by removing the participants who reported contradictory labels, such as positive and negative test results reported on the same day, positive and negative test results alternating in a short period, etc. This resulted in 118 longitudinal positive participants. In addition, we also checked the audio quality to remove the poor samples using Yamnet [28]: samples with background noise, clipped cough recordings due to larger amplitude than the maximum limit of mobile phones, etc. This resulted in 106 eligible positive participants.

For negative participants, similar selection criteria were used to identify eligible users. To generate a balanced dataset, we randomly selected 106 longitudinal negative participants from 447 participants.

In terms of sequence segmentation, a sliding window of length 5 (samples) and shift 1 (sample) was used to segment the long sequence into short sequences.

## Data augmentation

Three different data augmentation methods were employed:

- The first augmentation technique is the commonly used Gaussian noise augmentation, where randomly sampled Gaussian noise is added to the original waveform. To balance the positive and negative samples to some extent, the noise augmentation was applied three times to the original positive group and only 1 time to the negative group.
- The second augmentation method is our proposed sequence augmentation, where 5 random samples are collected from each participant's data pool and sorted in time order to generate new sequences. This increases the data size as well as enabling richer temporal dynamics. The same criterion of a maximum of 14 days between two adjacent recordings was applied for augmented sequences.
- The last augmentation method is the time inverse augmentation. Most of the reported sequences are consistently positive or negative, with only a limited number of samples reporting the transition from positive (negative) to negative (positive). The model might not effectively capture these transitions due to the limited data. Therefore, the segmented sequences were further time inverted during the training stage to enrich the dynamic changes.

All three augmentation techniques aided the model development by providing enough data, guaranteeing effective temporal dynamics, and increasing the various disease progression dynamics.

## Model architecture

### Feature extraction

The spectrograms were computed for 0.96 seconds for one recording, thus a series of spectrograms of each recording is converted to the same number of 128-dimensional feature vectors using *VGGish*. An average pooling layer is proposed to aggregate all vectors within one audio recording into one global latent feature vector. The global latent feature vectors obtained from each modality are concatenated to form a multi-model feature vector. Due to the limited size of the data, the pre-trained network *VGGish* originally optimised for acoustic event detection [28] is frozen and not trained.

### Irregular time intervals

The audio recordings for each participant are reported at irregular time intervals, where the days between consecutive audio recordings are not consistent. This is not compatible with GRUs modelling. Therefore, we assume any missing recording is the same as the last recording and use forward imputation. This is carried out for each day, and employed for the feature embeddings after the *VGGish* and pooling layers as:

| $\boldsymbol{x}_{t^{'}}=\boldsymbol{x}_{t}, t^{'}\in[1, T]$ | (1) |
| --- | --- |

where $t<t^{'}.$ $t^{'}$ can be any missing day and $t$ represents the days with recordings. In addition, we assume that the influence of the feature embeddings only has impact in a certain temporal context, and will fade away over time if the features have been missing for a long time. Therefore, a decay mechanism is designed for the feature embeddings, by incorporating the decaying factor in the additional feature dimension as:

| $\delta_{t}={0.75}^{\Delta t}$ | (2) |
| --- | --- |
| $\Delta t=t^{'}-t$ | (3) |
| ${\hat{\boldsymbol{x}}}_{t}=\left[ \boldsymbol{x}_{t}, \delta_{t} \right], t\in[1, T]$ | (4) |

where $\delta_{t}$ is the decaying factor, which is 1 for the present audio features and exponentially decaying for missing features. The final feature representation ${\hat{\boldsymbol{x}}}_{t}$ concatenates the original embeddings and the decaying factor.

### Sequential modelling

One GRUs layer and one dense layer are cascaded and served as the COVID-19 prediction layers. 64 neurons are adopted for GRUs layer and 2 neurons for dense layers. Softmax is used as the activation function, generating the probability scores for positive and negative detection. The probability scores are i) categorized into binary outputs of positive and negative; and ii) adopted directly examining the probabilities of positive over time. These correspond to the two different tasks respectively (cf. Figure 2).

### Multi-task learning

The subnetwork for language recognition in the multi-task learning framework consists of one dense layer (8 neurons) with softmax activation function, which takes the GRU outputs as the input and generate the language outputs. During the training phase, a reverse gradient layer is used to eliminate the language difference, with the loss as:

| $L=\alpha L_{\lambda_{1}}+\left( 1-\alpha\right)L_{\lambda_{2}}$ | (5) |
| --- | --- |

where $L$ is the final loss, and $L_{\lambda_{1}}$and $L_{\lambda_{2}}$represent the weighted cross-entropy loss for COVID-19 detection and focal loss for language recognition respectively. $\alpha$ is the scaling parameter that controls the balance between two subtasks.

## Model Training and Evaluation

### Training

During the training process, all the model parameters were optimised on the development set and validated in the test set. During training, an Adam optimizer was used. The learning rate and momentum was set to be 1e−4 and 0.90, respectively. Early stopping was also used as no increase of AUC-ROC was observed in 5 successive epochs. Weighted cross-entropy was used as the loss for COVID-19 detection, with weight optimized in [1,5] with a step size of 1. Focal loss was used for language detection with $\gamma=2$. For the multi-task learning, the weight $\alpha$ was optimized within [0.5,1.0] with an interval of 0.1. Tensorflow 2.0 was used for the model development.

### Evaluation metrics

The Point-Biserial Correlation Coefficient $\gamma_{pb}$ can be computed as:

| $\gamma_{pb}=\frac{\mu_{1}-\mu_{0}}{s_{n}} \sqrt{\frac{n_{1}n_{0}}{n^{2}}}$ | (6) |
| --- | --- |

Here $\mu_{1}$ and $\mu_{0}$ are the mean values of the predicted probabilities for the positive and negative samples of the participant, $s_{n}$ is the standard deviation of the predicted probabilities for all the samples of the participant. $n_{1}$ and $n_{0}$ are the numbers of samples in the positive and negative groups of the participant respectively, while $n$ is the total number $n=n_{1}+n_{2}$. A higher $\gamma_{pb}$ indicates a stronger correlation, thus a better disease progression trajectory prediction.

For the participants who reported positive and negative test results consistently over the clinical course, the ratio $\gamma$ is computed as:

| $\gamma= \frac{N_{i}}{N}$ | (7) |
| --- | --- |

where $N_{i}$and $N$ are the correctly predicted samples and the total number of samples of each individual.
