# Supplementary material for: Exploring Longitudinal Cough, Breath, and Voice Data for COVID-19 Progression Prediction via Sequential Deep Learning: Model Development and Validation
Source: J Med Internet Res. 2022 Jun 21;24(6):e37004. doi: 10.2196/37004 (PMC9217153; doi:10.2196/37004)
Supplement: Multimedia Appendix 2 [file jmir_v24i6e37004_app2.docx]

# Multimedia Appendix 2


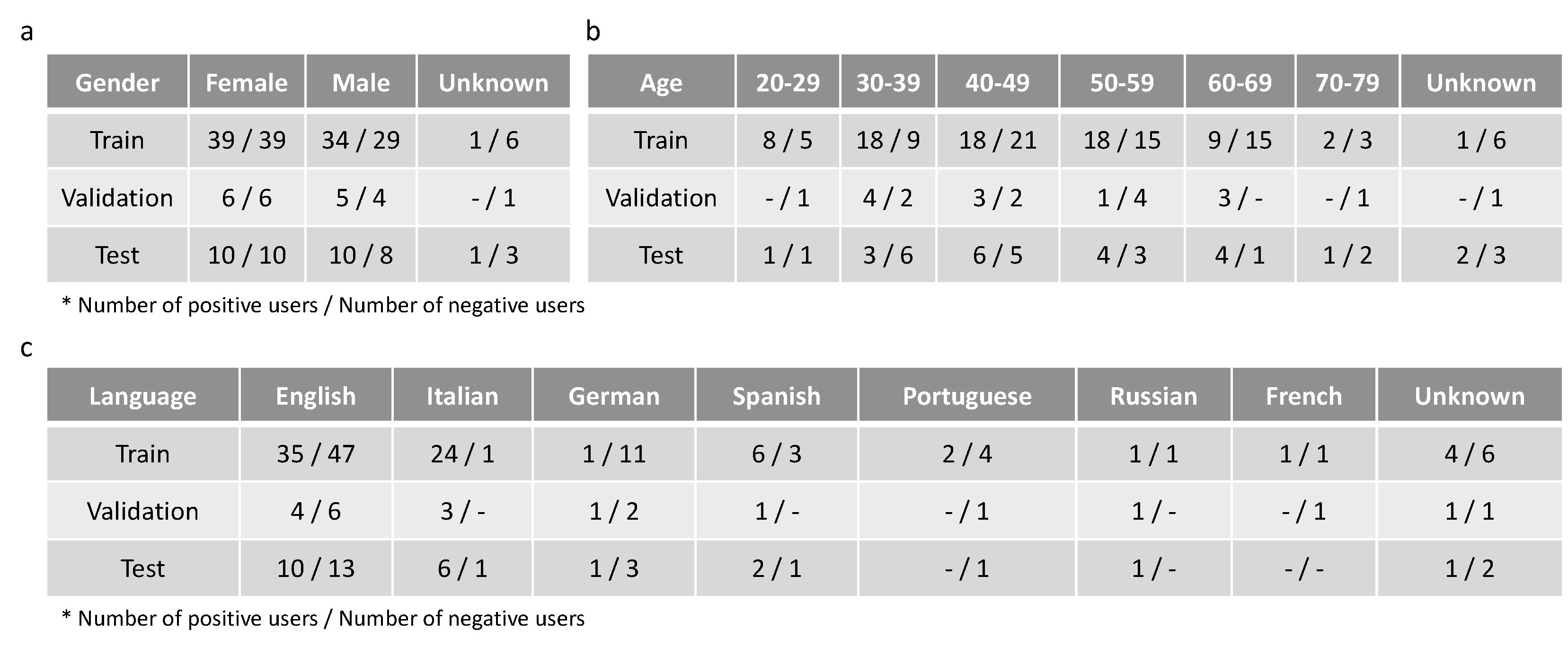


Figure A1. Data statistics in the training, validation, and test partitions in terms of gender, age, and language. a, Gender; b, Age; c, Language. Gender and age are relatively balanced for three partitions as well as for positive and negative groups. Language is balanced for three data partitions, but still unbalanced within each partition between positive and negative participants.
