# Supplementary material for: Exploring Longitudinal Cough, Breath, and Voice Data for COVID-19 Progression Prediction via Sequential Deep Learning: Model Development and Validation
Source: J Med Internet Res. 2022 Jun 21;24(6):e37004. doi: 10.2196/37004 (PMC9217153; doi:10.2196/37004)
Supplement: Multimedia Appendix 3 [file jmir_v24i6e37004_app3.docx]

# Multimedia Appendix 3


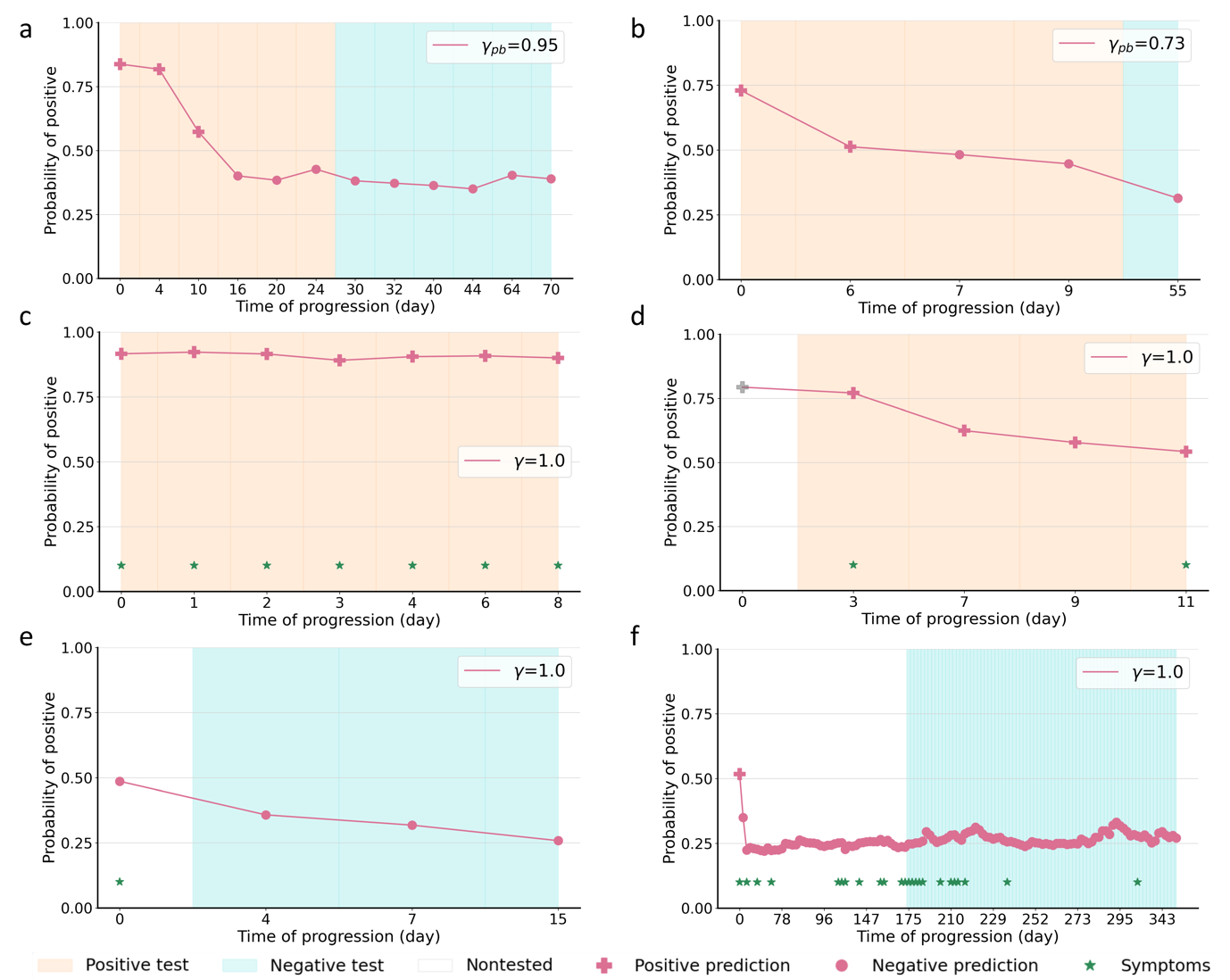


Figure A3. Examples of strong disease progression predictions. a and b, Two recovery participants; c and d, Two positive participants P6 and P7 reporting consistent positive test results; e and f, Two negative participants. Wrong prediction at day 0 for participant in (f) is possibly due to the limited audio dynamics. It should be noted that some of the audio recordings are not provided with the test results, labeled with the white shaded area.
