# Supplementary material for: Exploring Longitudinal Cough, Breath, and Voice Data for COVID-19 Progression Prediction via Sequential Deep Learning: Model Development and Validation
Source: J Med Internet Res. 2022 Jun 21;24(6):e37004. doi: 10.2196/37004 (PMC9217153; doi:10.2196/37004)
Supplement: Multimedia Appendix 4 [file jmir_v24i6e37004_app4.docx]

# Multimedia Appendix 4


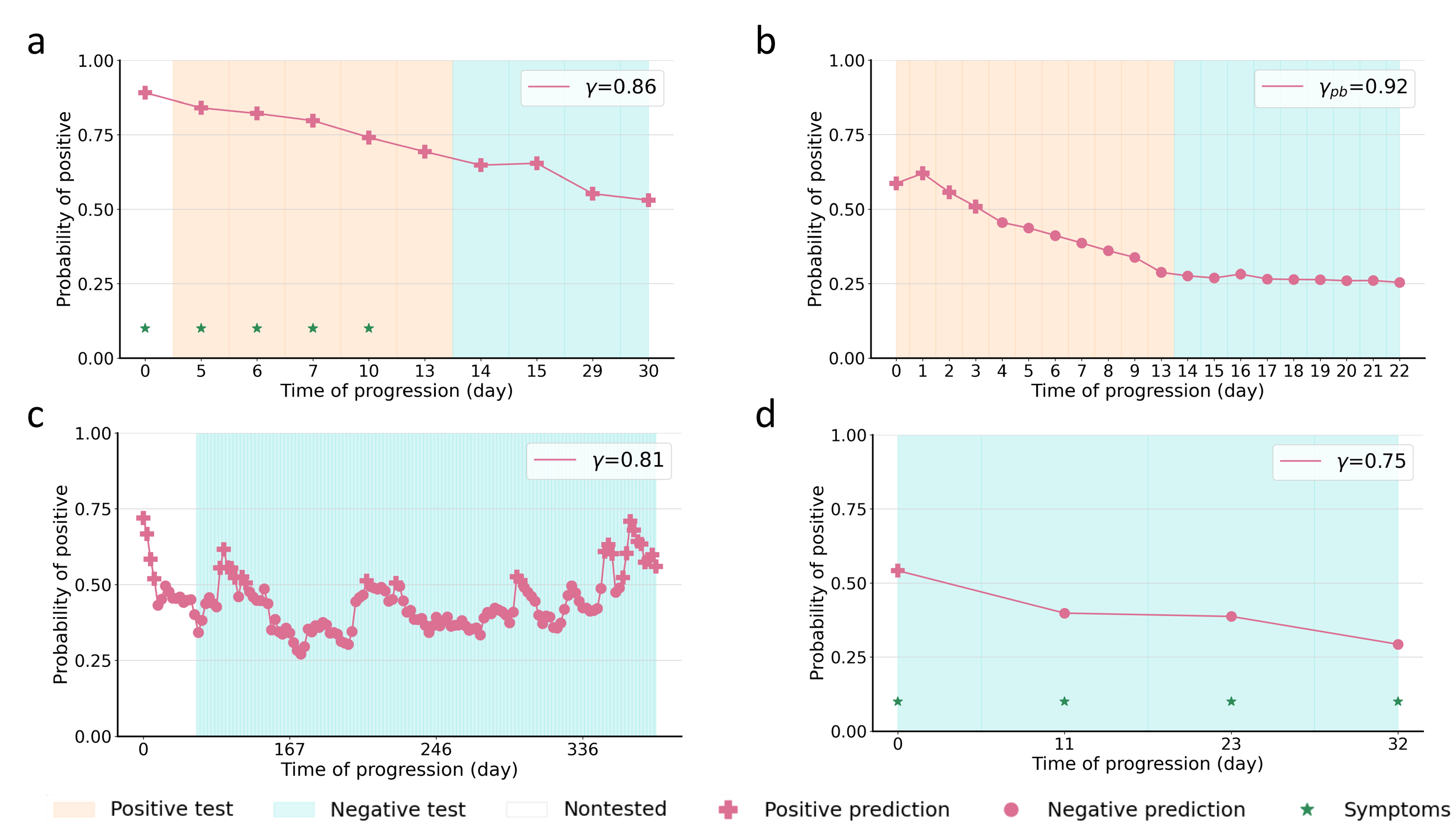


Figure A4. Examples of weak disease progression predictions. a and b, Two recovery participants. The predicted recovery trend is observed, while the categorized positive and negative predictions are not exactly matching the test results, which implies the potential personalisation. c and d, Two negative participants. Despite the accuracy of $\gamma=0.81$ for participant c, the predictions fluctuate between positive and negative predictions, which suggests the possible solution of personalisation. Participant d was predicted positive for the 1st point with the probability of 0.55, which is possibly due to the inadequate audio dynamics. The predictions were predicted correctly after capturing richer audio dynamics by using a longer sequence.
