# Supplementary material for: Exploring Longitudinal Cough, Breath, and Voice Data for COVID-19 Progression Prediction via Sequential Deep Learning: Model Development and Validation
Source: J Med Internet Res. 2022 Jun 21;24(6):e37004. doi: 10.2196/37004 (PMC9217153; doi:10.2196/37004)
Supplement: Multimedia Appendix 5 [file jmir_v24i6e37004_app5.docx]

# Multimedia Appendix 5


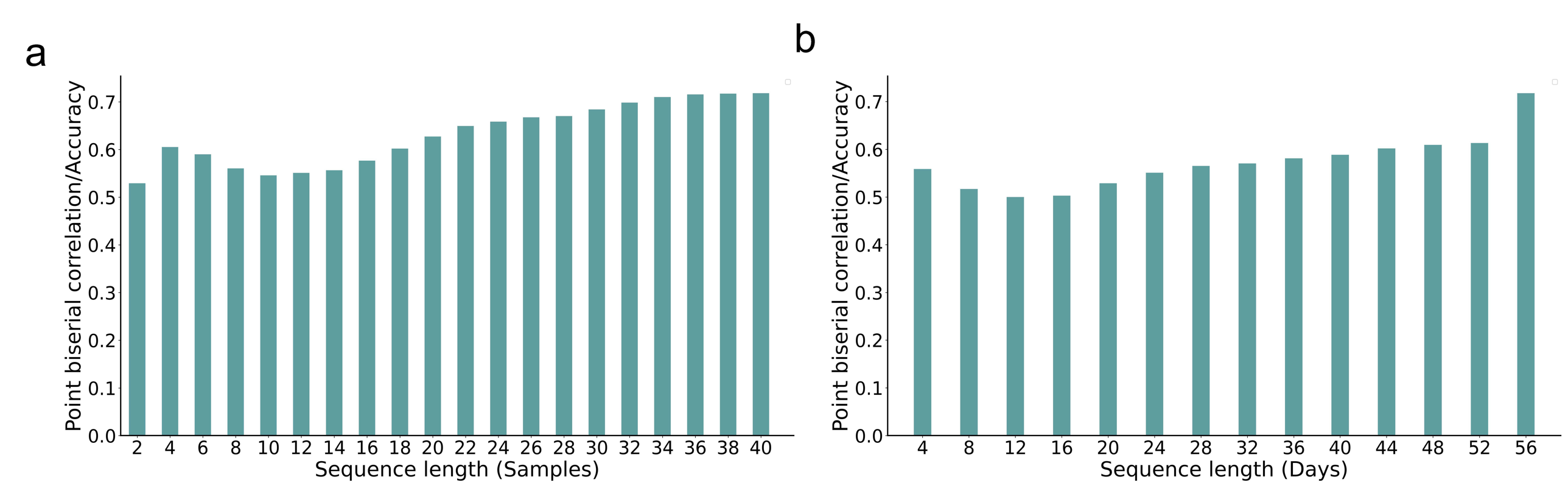


Figure A2. The performance of COVID-19 detection with different sequence length in (a) samples and (b) days. X axis indicates the maximum sequence length of the subgroup, and y axis shows the accuracy for the sequences shorter than the maximum length.
